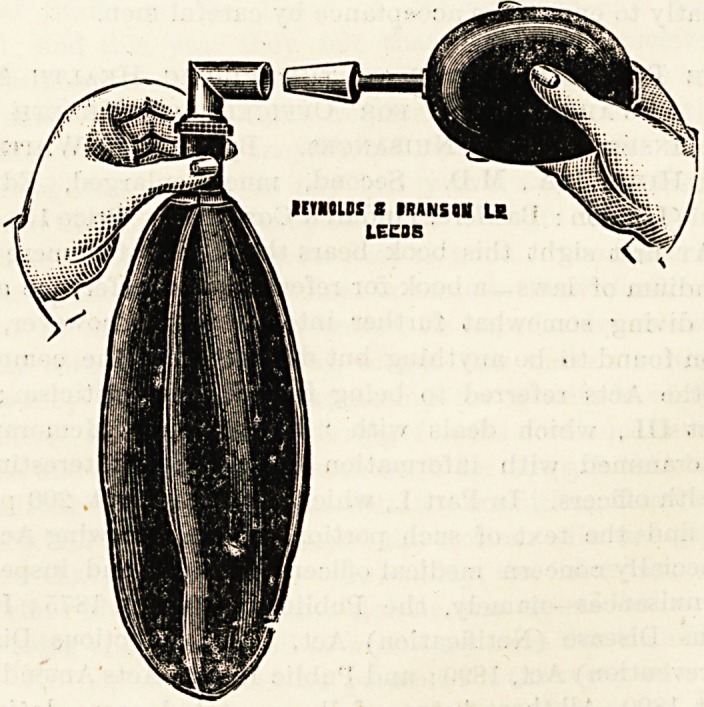# New Appliances and Things Medical

**Published:** 1901-08-03

**Authors:** 


### NEW APPLIANCES AND THINGS MEDICAL.

[We shall be glad to receive, at our Office, 28 & 29 Southampton Street, Strand, London, W.C., from the manufacturers, specimens of all new preparations and appliances which may be brought out from time to time.]

### LAIT LAROLA.

(M. BEETHAM AND SONS, CHELTENHAM.)

LAIT LAROLA is a very useful and cooling application for the skin; its special properties will be most appreciated by those who suffer from sun burn and roughness of the skin when exposed to drying winds. It contains no harmful ingredient, and, therefore, may be safely employed in the nursery. It is pleasantly scented and highly emollient.

### WRIGHT'S COAL-TAR SOAP, LIQUOR CARBONIS DETERGENS, AND OTHER PREPARATIONS.

(WRIGHT, LAYMAN AND UMNEY, LIMITED, 48 SOUTHWARK STREET, LONDON, S.E.)

THIS well-known soap contains the valuable antiseptic bodies which are derivatives of coal tar. The basis of the soap is neutral in reaction, and by a skilful pharmacy the characteristic odour of the coal-tar antiseptics is pleasantly disguised by a suitable perfume. As an antiseptic soap for toilet purposes we thoroughly recommend it. Wright's liquor carbonis detergens, which is an alcoholic solution of certain of the derivatives of coal tar, which possess powerful antiseptic properties, is one of the finest and most powerful germicides in the market. In the last issue of the British Pharmacopœia a somewhat similar preparation was included in the list of additions, which, though highly complimentary to the proprietors of the original liquor, is by no means of the same chemical constitution. We therefore recommend all practitioners who prescribe this valuable antiseptic to order "Wright's" liquor. The sapo-carbonis detergent dentifrice contains 10 per cent. of coal-tar soap in addition to other innocuous antiseptics, and is a pleasant, non-poisonous, and cleansing preparation. We recommend it as a useful dentifrice for everyday use. Wright's coal-tar shaving soap is a safe and pleasant preparation.

### NEW INFLATOR FOR ETHER BAG.

(REYNOLDS AND BRANSON, 13 BRIGGATE, LEEDS.)

THIS inflator effectively performs the duty of filling the bag of a Clover's inhaler with air. A duty which is often carried out by the anæsthetist's lungs, a by no means convenient or desirable operation.

The ether bag must be inflated at the commencement and sometimes during an operation; it is very handy to have a simple apparatus to carry out the necessary proceeding; it is a wonder that the idea has been left to the present century for Mr. Herbert J. Robson to carry into effect.

## Figures and Tables

**Figure f1:**